# Relationship between Social Support Networks and Physical Functioning in Older Community-Dwelling Mexicans

**DOI:** 10.3390/ijerph14090993

**Published:** 2017-08-31

**Authors:** Víctor Manuel Mendoza-Núñez, Fabiola González-Mantilla, Elsa Correa-Muñoz, Raquel Retana-Ugalde

**Affiliations:** 1 Unidad de Investigación en Gerontología, Facultad de Estudios Superiores Zaragoza, Universidad Nacional Autónoma de México (UNAM), Guelatao N° 66, Col. Ejército de Oriente, 09230 Mexico City, Mexico; menuvi05@yahoo.com.mx (E.C.-M.); retanara@unam.mx (R.R.-U.); 2 Maestría en Enfermería, Universidad Nacional Autónoma de México, CP 04510 Mexico City, Mexico; mendovic1956@gmail.com

**Keywords:** social support networks, physical functioning, healthy ageing, Mexican older people

## Abstract

Some studies have demonstrated the relationship between social support networks (SSNs) and health status. In this sense, it has been considered that physical and mental functioning is a key indicator of the health in the age people. The aim of this study was to determine the association between social support networks and physical functioning. A cross-sectional study was carried out including a convenience sample of 150 older community-dwelling Mexicans. We assessed the familial, extra-familial and institutional SSNs; social contacts; the activities of daily living (ADL); the instrumental activities of daily living (IADLs); and physical functioning task (PFT) performance among study participants. Of the 150 older subjects, 53 reported living alone (35%), 113 (75%) reported having few SSNs, and 37 (25%) reported having enough SSNs. Persons with few familial SSNs were at increased odds of demonstrating dependence in at least one of the ADL (OR = 3.25, 95% CI 1.06–9.92, *p* < 0.05). Likewise, persons with few institutional SSNs and few social contacts were at increased odds of demonstrating dependence in at least one of the IADL (OR = 6.96, 95% CI 1.57–30.7, *p* < 0.01; OR = 5.02, 95% CI 1.44–17.5, *p* < 0.01, respectively). We also observed that having few extra-familial SSNs and few social contacts were the main risk factors for PFT dependence, with ORs of 3.70 (95% CI 1.21–11.2, *p* < 0.05) and 3.85 (95% CI 1.10–13.5, *p* < 0.05), respectively. Our findings suggest that having few SSNs could be a significant risk factor for reduced physical functioning in older adults.

## 1. Introduction

Healthy aging is one of the main goals of governments worldwide in public health programs. However, no consensus has been established regarding what this concept might comprise or how it may be defined or measured. Furthermore, healthy ageing is often used to describe a positive disease-free state and distinguish between healthy and unhealthy individuals. This definition is problematic in persons of older age because many individuals may have one or more health conditions that are well controlled and have little influence on their ability to function [1]. For this reason, the WHO has defined healthy ageing as “the process of developing and maintaining the functional ability that enables well-being in older age”; additionally, the WHO has defined “functional ability as the health-related attributes that enable people to be and to do what they have reason to value. It is made up of the intrinsic capacity of the individual, relevant environmental characteristics and the interactions between the individual and these characteristics” [1].

Physical functioning is considered the ability of a person to be independent in his or her personal care and active social participation. This ability is determinant of the prevention and control of the chronic diseases that are more prevalent in old age, as independence favours self-care, including that associated with health [1]. The determination of whether a person is independent can be based on assessments of physical functioning tasks (PFT), which are linked to the basic activities of daily living (ADL), and the instrumental activities of daily living (IADL). This physical functioning capacity can potentially enable active social participation [2,3,4].

Some studies have demonstrated the usefulness of nutrition and physical exercise in efforts to achieve healthy ageing, considering physical and mental functioning to be a key element of these health behaviours [5,6,7]. However, studies have also reported that social support networks (SSNs) can have a significant effect on the health status of the older adults [8,9,10]. In this sense, social support promotes health because it facilitates healthier behaviours such as exercise, eating correcly, and not smoking, as well as greater adherence to medical regimens [11,12].

Social support networks are defined as connections and contacts with people through which emotional, informational and instrumental support is received. Informal networks, which are shaped by family members, relatives and friends, are considered to be an emotional bond that determines mutual support and reciprocity [13]. However, formal networks include community groups and organized civil society associations whose members adhere to the group consciousness through a membership that establishes commitments, guidelines and work rules and a hierarchical organization that is established by the members themselves. In this regard, seven psychosocial mechanisms have been proposed to explain the positive effect of social support networks on physical and mental health: “(i) social influence/social comparison; (ii) social control; (iii) behavioural guidance, purpose, and meaning (mattering); (iv) self-esteem; (v) sense of control or mastery; (vi) belonging and companionship; and (vii) perceived social support” [12,14].

Several studies have shown a positive effect of SSNs on health and physical functioning [9,15,16]. However, social networks per sedo not have a positive effect on health, well-being and quality of life, since social ties can also have a negative influence in promoting harmful lifestyles and/or being a source of stressful situations [13]. Besides this, it has also been observed that they can have an ambivalent effect [17].

In Mexico in 2015, 10.4% of the total population (12.4 million) was older (≥60 years), and demographic projections indicate that, by 2050, 27.7% of the Mexican population will be aged 60 years or older. In addition, the average life expectancy at age 60 is 22 years [18]. This demographic situation has legal, social and economic implications and, above all, health risks. In this sense, the National Health and Nutrition Examination Survey reported that the prevalence of arterial hypertension and type 2 diabetes mellitus in people over 60 years of age is more than 50% and more than 20% respectively. Likewise, more than 25% of all older adults have some limitation in the physical functionality [19]. In this sense, the family is the main source of the SSNs, so it has been observed that the majority of older people live in extended family households, with relatives such as married children. Others live in nuclear households with children who are still unmarried and dependent on their parents, and few live alone [10,20].

In this context, the SSNs especially the family, and community self-help groups framed in a model of active aging, could be a determinant capital social for achieving healthy aging [21].

Therefore, the aim of this study was to determine the association between social support networks and physical functioning in older community-dwelling Mexicans.

## 2. Methods

### 2.1. Subjects and Design

A cross-sectional study was carried out including a convenience sample of 150 older people (≥60 years) who agreed to participate in the self-care and mutual help community program. The Ethics Committee of the Universidad Nacional Autónoma de México (UNAM) Zaragoza Campus approved the research protocol for this study (PAPIME PE305516). The subjects agreed to participate in the study after providing informed consent. Informative brochures were distributed in the community specifying the objectives of the study and admission criteria: (i) interest in participating in the study; (ii) level of literacy (3–9 years of education); and (iii) absence of handicapping illnesses or serious visual or auditory disabilities. The subjects were community-dwelling elderly people living in Mexico City Metropolitan Area for 10 years or more, with a low income (≤US $300.00 per month).

### 2.2. Social Support Networks for Older People Scale (SSN-Older)

The participants’ social support networks were assessed by administering the Spanish version of the SSN-Older scale, which has been validated in older Mexican people. This questionnaire assessed the following three social support networks: familial SSN, including a participant’s wife or husband, brothers, sisters, cousins, sons, daughters, grandsons, granddaughters, nephews and nieces; extra-familial SSN including a participant’s friends, partners, and fellow community group members; and institutional SSN, including a participant’s health care institutions and social care institutions. The SSN-Older scale includes 54 network-related questions: (i) familial SSN, 36 questions, with a maximum total score of 57, including scores relating to social contact, support received, and satisfaction with support received; (ii) extra-familial SSN, nine questions, with a maximum total score of 38, including scores relating to social contact, support received, and satisfaction with support received; and (iii) institutional SSN, nine questions, with a maximum total score of 19, including scores relating to social contact, support received, and satisfaction with support received. Some items on the questionnaire are scored using Likert scale scores for social contact: 0, never; 1, rarely (less than once a month); 2, sometimes (once or twice a month); and 3, often (at least once a week). SSN-Older also assesses a participant’s satisfaction with his or her social support networks and number of social contacts with a dichotomous score: yes (1) or no (0). The reliability of the instrument was alpha = 0.93 [22].

For the purposes of this study, the maximum and minimum potential real score of the social support networks of the population was considered. It is this sense, the level for sufficient social networks (ESN) was established as scores equal or greater than the 75th percentile and few social networks (FSN) when scores were equal or below the 25th percentile.

**Scores:**

Familial SSN: FSN ≤ 18; ESN ≥ 36

Extra-familial SSN: FSN ≤ 11; ESN ≥ 28

Institutional SSN: FSN ≤ 2; ESN ≥ 10

Social contacts: FSN ≤ 9; ESN ≥ 13

Support received: ≤7; ESN ≥ 14

Satisfaction with support received: ≤17; ESN ≥ 44

### 2.3. Activities of Daily Living (ADLs)

The Barthel Index was used for measuring the ADLs. The tool may be used to assess basic activities of daily living in older adults, including the measurement of functional levels of self-care and mobility and the ability to feed and groom oneself, bathe, go to the toilet, walk or use a wheelchair, climb stairs, and control one’s bowel and bladder. Scores on the ADL may vary from 0 to 100 in accordance with the older adult’s ability to perform the daily activities on his or her own. A score of 0 indicates complete dependency in the activities of daily living, whereas as score of 100 indicates complete independence in the activities of daily living [2].

### 2.4. Instrumental Activities of Daily Living (IADLs)

The following IADLs were measured based on self-rated ability: telephone use, transportation out of walking distance, shopping, preparing meals, doing housework, managing money, and taking medication. Each functional item can be categorized as independent, partially dependent, or completely dependent. We consider a subject as independent when her or she reported being independent in all activities and defined dependent subjects as those who reported being partially or completely dependent in the performance of one or more activities [3].

### 2.5. Physical Functioning Tasks (PFT)

Physical functioning tasks were measured using the Nagi scale, on which the following abilities are considered: (i) the ability to stand for 15 min (self-rated); (ii) sit down for 1 h (self-rated); (iii) crouch, squat, kneel, and bow (demonstrative); (iv) raise arms under and over shoulder level (demonstrative); (v) grasp with small things with one’s fingers (demonstrative); (vi) lift a heavy object weighing 1 to 5 kg (demonstrative); (vii) lift a heavy object weighing 5 to 10 kg (demonstrative); and (viii) push or pull a large object (self-rated). Each functional item can be categorized as independent, partially dependent, or completely dependent [4].

### 2.6. Statistical Analyses

The data were processed using the SPSS V. 20.0 standard statistical software package (IBM SPSS Statistics, Armonk, NY, USA). The descriptive statistics included means, standard deviations (SDs), and percentages. In order to establish the cutoffs of the few and enough social networks, the values of the 25th and 75th percentiles of the scores of the social support networks for older people scale were obtained. Results were analyzed using linear correlation analyses. Also were performed odds ratio (OR) of logistic regression analysis with 95% confidence interval (CI). A *p*-value < 0.05 was considered.

## 3. Results

Of the 150 persons ≥ 60 years who participated in the study, 99 were women (66%) and 51 were men (34%); the mean age of participants was 69 ± 7 years. With respect to coexistence with a family member, 53 people reported living alone (35%), and 97 people reported living with a relative, mainly a spouse. Regarding the distribution of social support networks, 113 people (75%) reported few networks and 37 (25%) had enough social networks (Table 1).

Persons with few familial SSNs were at increased odds of demonstrating dependence in at least one ADL (OR = 3.25, 95% CI 1.06–9.92, *p* < 0.05). Similarly, with few extra-familial SSNs and few social contacts were at increased odds of demonstrating dependence in at least one ADL (OR = 2.64, 95% CI 1.0–7.36, *p* < 0.05; OR = 3.70, 95% CI 1.0–1.3, *p* < 0.05, respectively) (Table 2).

Likewise, in the assessment of the association between SSNs and IADL, persons with few institutional SSNs and few social contacts were at increased odds of demonstrating dependence in at least one IADL (OR = 6.96, 95% CI 1.57–30.7, *p* < 0.01; OR = 5.02, 95% CI 1.44–17.5, *p* < 0.01, respectively); additionally, we observed that having few familial and extra-familial SSNs negatively influenced IADL (Table 3).

We also observed that having few extra-familial SSNs and few social contacts we the main risk factors for demonstrating dependency on the physical functioning tasks (PFTs), with ORs of 3.70 (95% CI 1.21–11.2, *p* < 0.05) and 3.85 (95% CI 1.10–13.5, *p* < 0.05), respectively (Table 4).

Regarding the correlation between SSN-Older score and scores on the ADL, IADL and physical capacity scales, statistically significant associations were found between the familial SSN subscale score and ADL scale (r = 0.24, *p* < 0.01), IADL scale (r = 0.34, *p* < 0.001) and PFT scale scores (r = 0.31, *p* < 0.001). We also identified statistically significant correlations between the extra-familial SSN subscale score and with the ADL scale (r = 0.23, *p* < 0.01), IDL scale (r = 0.41, *p* < 0.001) and PFT scale scores (r = 0.44, *p* < 0.001). In addition, statistically significant correlations were found between the score on the subscale for satisfaction with support received and ADL scale (r = 0.23, *p* < 0.01), IADL scale (r = 0.38, *p* < 0.001) and PFT scale scores (r = 0.40, *p* < 0.001). We also found statistically significant correlations between the number of social contacts and the scores on the ADL scale (r = 0.34, *p* < 0.001), IADL scale (r = 0.45, *p* < 0.001) and PFT scale (r = 0.41, *p* < 0.001) (Table 5).

## 4. Discussion

The types of networks reflect information about structure, function and quality [23]. These characteristics have a significant influence on well-being, and the physical and/or mental health of older people [24,25]. In this sense, the magnitude of the SSNs could have a significant effect on health and physical functioning; however, there is no criterion that allows the establishment of an ideal cut-off point. For this reason, for the purposes of our study, two categories were established (few and enough), considering the maximum and minimum potential real score of the SSNs, considering the25th and 75thpercentiles, respectively. The results of our study showed that the majority of older adults (75%) had few social support networks. This social situation represents a possible risk factor for achieving healthy ageing. In this regard, some studies have shown that having sufficient social support networks promotes self-care, as it is associated with health, thereby preventing and controlling the prevalence of some chronic diseases in the community [8,26,27].

In this sense, we found that having few familial and extra-familial SSNs and few social contacts could be risk factors for demonstrating dependence in the activities of daily living, and a high percentage (35%) of the older adults in this study lived alone. Previous studies have suggested that less social support, few social networks, and negative social interactions may be linked to depression, poorer immune functioning, an increased incidence of disease, and higher mortality [28,29]. Likewise, studies have also shown intervention programmes that strengthen social support networks to have a positive effect on physical and mental health as well as functioning in older adults living alone [30,31].

The instrumental activities of daily living, such as telephone use, transportation out of walking distance, shopping, and managing money, allow for greater socialization among older adults. Likewise, greater socialization promotes the maintenance of IADLs. For this reason, in our study, having few institutional SSNs and few social contacts could be risk factors for demonstrating dependency in the IADLs. In this regard, studies have shown that institutional programs increase social contacts, promotes physical activity, improve psychosocial perceptions and increase longevity [32,33].

The physical functioning tasks measured with the Nagi scale allowed us to infer the functional capacity of older adults as it pertains to active participation in social and community programs, as well as older adults’ ability to work and travel independently [4]. In this study, we found that having few extra-familial SSNs, few social contacts and a low level of satisfaction with the support received could be risk factors for demonstrating dependency in at least one of the abovementioned tasks and may serve as limitations to active participation in ageing programs. In accordance with these findings, similar results have been observed in populations from different cultures, among which we can highlight studies carried out in older adults who were Mexican–American, African–American and European, regarding the relationship of social networks with mortality risks, physical health and life satisfaction [34,35,36].

## 5. Limitations

The design of this study must be considered as one of the main limitations, as it was cross-sectional and exploratory, the sample was taken at convenience, and its size was not representative. Therefore, our findings cannot be generalized; however, the relationship observed between SSNs and physical functioning in older adults supports the hypothesis that few SSNs could be a risk factor for the loss of physical functioning in older people.

## 6. Conclusions

Our findings suggest a possible positive relationship between social support networks and physical functioning in older community-dwelling Mexicans, especially familial-SSN and social contacts, due probably to the sociocultural aspects of our population. In this sense, solidarity and intergenerational family support in Mexican society is still maintained [37]; however, this situation is not generalized, as in the present study we observed that the majority (75%) of the population studied reported few social networks. For this reason, it is necessary to promote programs that generate and strengthen social support networks, which recognize the social capital represented by the family and the community support among the elderly, framed in public health programs related to healthy aging.

## Figures and Tables

**Table 1:** Socio-demographic characteristics and social support networks.

Variable	*n* = 150	%
Sex		
Women	99	66
Men	51	34
Age (years)	69 ± 7	
Status marital		
Married	70	47
Single or widowed	80	53
Cohabitation		
Spouse	70	47
Children	11	7
Relatives	16	11
Alone	53	35
Enough social networks	37	25
Few social networks	113	75

**Table 2:** Social support networks as a risk factor for activities of daily living (ADL) dependence.

Variable	ESN *n* = (%)	FSN *n* = (%)	OR	95% CI	*p*-Value
Familial SSN					
Independent	31 (89)	81 (70)	3.25	1.06–9.92	0.03
Dependent	4 (11)	34 (30)			
Extra-familial SSN					
Independent	32 (87)	80 (71)	2.64	1.0–7.36	0.04
Dependent	5 (13)	33 (29)			
Institutional support					
Independent	24 (89)	88 (72)	3.18	0.90–11.2	0.06
Dependent	3 (11)	35 (28)			
Social contacts					
Independent	27 (90)	85 (71)	3.70	1.0–1.3	0.03
Dependent	3 (10)	35 (29)			
Support received					
Independent	28 (88)	84 (71)	2.83	0.92–8.69	0.06
Dependent	4 (12)	34 (29)			
Satisfaction with support received					
Independent	31 (86)	81 (71)	2.52	0.90–7.05	0.07
Dependent	5 (14)	33 (29)			

ADL, activities of daily living; ESN, enough social networks; FSN, few social networks; familial SSN, familial social support networks; extra-familial SSN, extra-familial social support networks; OR, odds ratio; 95% CI, 95% confidence interval.

**Table 3:** Social support networks as a risk factor for instrumental activities of daily living (IADL) dependence.

Variable	ESN *n* = (%)	FSN *n* = (%)	OR	95% CI	*p*-Value
Familial SSN					
Independent	30 (86)	74 (64)	3.32	1.19–9.92	0.01
Dependent	5 (14)	41 (36)			
Extra-familial SSN					
Independent	31 (84)	73 (65)	2.83	1.08–7.36	0.02
Dependent	6 (16)	40 (35)			
Institutional support					
Independent	25 (93)	79 (64)	6.96	1.57–30.7	0.004
Dependent	2 (7)	44 (36)			
Social contacts					
Independent	27 (90)	77 (64)	5.02	1.44–17.5	0.006
Dependent	3 (10)	43 (36)			
Support received					
Independent	26 (81)	78 (66)	2.22	0.84–5.83	0.09
Dependent	6 (19)	40 (34)			
Satisfaction with support received					
Independent	30 (83)	74 (65)	2.70	1.03–7.03	0.03
Dependent	6 (17)	40 (35)			

IADL, instrumental activities of daily living; ESN, enough social networks; FSN, few social networks; Familial SSN, familial social support networks; Extra-familial SSN, extra-familial social support networks; OR, odds ratio; 95% CI, 95% confidence interval.

**Table 4:** Social support networks as a risk factor for physical functioning tasks (PFT) dependence.

Variable	ESN *n* = (%)	FSN *n* = (%)	OR	95% CI	*p*-Value
Familial SSN					
Independent	29 (83)	82 (71)	1.94	0.739–5.11	0.10
Dependent	6 (17)	33 (29)			
Extra-familial SSN					
Independent	33 (89)	78 (69)	3.70	1.21–11.2	0.01
Dependent	4 (11)	35 (31)			
Institutional support					
Independent	23 (85)	88 (72)	2.28	0.737–7.09	0.10
Dependent	4 (15)	35 (28)			
Social contacts					
Independent	27 (90)	84 (70)	3.85	1.10–13.5	0.02
Dependent	3 (10)	36 (30)			
Support received					
Independent	28 (88)	83 (70)	2.95	1.0–9.04	0.04
Dependent	4 (12)	35 (30)			
Satisfaction with support received					
Independent	29 (81)	82 (72)	1.61	0.64–4.00	0.30
Dependent	7 (19)	32 (28)			

PFT, physical functioning tasks; ESN, enough social networks; FSN, few social networks; Familial SSN, familial social support networks; Extra-familial SSN, extra-familial social support networks; OR, odds ratio; 95% CI, 95% confidence interval.

**Table 5:** Correlation between social support networks for older people (SSN-Older) scores and scores for ADL, IADL and PFT.

Variable	ADL	IADL	PFT
	r	r	r
Familial SSN	0.242 **	0.342 ***	0.312 ***
Extra-familial SSN	0.231 **	0.410 ***	0.441 ***
Institutional support	0.138	0.168 *	0.188 *
Satisfacción de apoyo	0.238 **	0.389 ***	0.402 ***
Social contacts	0.341 **	0.457 ***	0.417 ***
Support received	0.265 ***	0.351 ***	0.378 ***

ADL, activities of daily living; IADL, instrumental activities of daily living; PFT, physical; functioning tasks. Pearson’s correlation:* *p* < 0.05, ** *p* < 0.01, *** *p* < 0.001.

## References

[1] World Health Organization (2015). World Report on Ageing and Health.

[2] Mahoney F.I., Barthel D. (1965). Functional evaluation: The barthel index. Md. State Med. J..

[3] Lawton M.P., Brody E.M. (1969). Assessment of older people: Self maintaining and instrumental activities of daily living. Gerontologist.

[4] Nagi S.E. (1976). An epidemiology of disability among adults in the United States. Milbank Memml. Fund Q..

[5] Bunout D., Barrera G., de la Maza P., Avendaño M., Gattas V., Petermann M., Hirsch S. (2001). The impact of nutritional supplementation and resistance training on the health functioning of free-living Chilean elders: Results of 18 months of follow-up. J. Nutr..

[6] Verdijk L.B., Jonkers R.A., Gleeson B.G., Beelen M., Meijer K., Savelberg H.H., Wodzig W.K., Dendale P., Van Loon L.J. (2009). Protein supplementation before and after exercise does not further augment skeletal muscle hypertrophy after resistance training in elderly men. Am. J. Clin. Nutr..

[7] Bemben M.G., Witten M.S., Carter J.M., Eliot K.A., Knehans A.W., Bemben D.A. (2010). The effects of supplementation with creatine and protein on muscle strength following a traditional resistance training program in middle-aged and older men. J. Nutr. Health Aging.

[8] Mendoza-Núñez V.M., Flores-Bello C., Correa-Muñoz E., Retana-Ugalde R., Ruiz-Ramos M. (2016). Relationship between social support networks and diabetes control and its impact on the quality of life in older community-dwelling Mexicans. Nutr. Hosp..

[9] Seeman T.E. (2000). Health promoting effects of friends and family on health outcomes in older adults. Am. J. Health Promot..

[10] De Vos S., Solís P., Montes de Oca V. (2004). Receipt of assistance and extended family residence among elderly men in Mexico. Int. J. Aging Hum. Dev..

[11] Thoits P.A. (2011). Mechanisms linking social ties and support to physical and mental health. J. Health Soc. Behav..

[12] Uchino B.N., Cacioppo J.T., Kiecolt-Glaser J.K. (1996). The relationship between social support and physiological processes: A review with emphasis on underlying mechanisms and implications for health. Psychol. Bull..

[13] Smith K.P., Christakis N.A. (2008). Social networks and health. Annu. Rev. Sociol..

[14] Uchino B.N. (2006). Social support and health: A review of physiological processes potentially underlying links to disease outcomes. J. Behav. Med..

[15] Ertel K.A., Glymour M.M., Berkman L.F. (2009). Social networks and health: A life course perspective integrating observational and experimental evidence. J. Soc. Pers. Relatsh..

[16] Unger J.B., McAvay G., Bruce M.L., Berkman L., Seeman T. (1999). Variation in the impact of social network characteristics on physical functioning in elderly persons: MacArthur studies of successful aging. J. Gerontol. B Psychol. Sci. Soc. Sci..

[17] Uchino B.N., Cawthon R.M., Smith T.W., Light K.C., McKenzie J., Carlisle M., Gunn H., Birmingham W., Bowen K. (2012). Social relationships and health: Is feeling positive, negative, or both (ambivalent) about your social ties related to telomeres?. Health Psychol..

[18] Instituto Nacional de Estadística y Geografía (2016). Estadísticas a Propósito Del Día Internacional De Las Personas De Edad (1 De Octubre).

[19] Gutiérrez J.P., Rivera-Dommarco J., Shamah-Levy T., Villalpando-Hernández S., Franco A., Cuevas-Nasu L., Romero-Martínez M., Hernández-Ávila M. (2013). Encuesta Nacional De Salud y Nutrición 2012, Resultados Nacionales.

[20] Robles L., Rizo Curiel G., Camarena García L.M., Cervantes Coles L., Gómez Medrano M.S., Siordia González M. (2000). Social network and social support among poor elderly ill in Guadalajara, Mexico. Cad. Saude Publ..

[21] Greenfield E.A. (2014). Community aging initiatives and social capital: Developing theories of change in the context of NORC Supportive Service Programs. J. Appl. Gerontol..

[22] Mendoza-Núñez V.M., Martínez-Maldonado M.L. (2009). Escala de redes de apoyo social para adultosmayores. González-Celis RAL.

[23] Fiori K.L., Smith J., Antonucci T.C. (2007). Social network types among older adults: A multidimensional approach. J. Gerontol. B Psychol. Sci. Soc. Sci..

[24] Heller T., Fisher D., Marks B., Hsieh K. (2014). Interventions to promote health: Crossing networksof intellectual and developmental disabilities and aging. Disabil. Health J..

[25] Li T., Zhang Y. (2015). Social network types and the health of older adults: Exploring reciprocal associations. Soc. Sci. Med..

[26] Huxhold O., Miche M., Schüz B. (2014). Benefits of having friends in older ages: Differential effects of informal social activities on well-being in middle-aged and older adults. J. Gerontol. B Psychol. Sci. Soc. Sci..

[27] Sánchez-Rodríguez M.A., Arronte-Rosales A., Mendoza-Núñez V.M. (2009). Effect of a self-care program on oxidative stress and cognitive function in an older Mexican urban-dwelling population. J. Nutr. Health Aging.

[28] Yang Y.C., McClintock M.K., Kozloski M., Li T. (2013). Social isolation and adult mortality: The role of chronic inflammation and sex differences. J. Health Soc. Behav..

[29] Nyqvist F., Forsman A.K., Giuntoli G., Cattan M. (2013). Social capital as a resource for mental well-being in older people: A systematic review. Aging Ment. Health.

[30] Palmer A.D., Newsom J.T., Rook K.S. (2016). How does difficulty communicating affect the social relationships of older adults? An exploration using data from a national survey. J. Commun. Disord..

[31] Health Quality Ontario (2008). Social isolation in community-dwelling seniors: An evidence-based analysis. Ont. Health Technol. Assess. Ser..

[32] Yang Y.C., Boen C., Gerken K., Li T., Schorpp K., Harris K.M. (2016). Social relationships and physiological determinants of longevity across the human life span. Proc. Natl. Acad. Sci. USA.

[33] Barnett A., Cerin E., Zhang C.J., Sit C.H., Johnston J.M., Cheung M.M.C., Lee R.S.Y. (2016). Associations between the neighbourhood environment characteristics and physical activity in older adults with specific types of chronic conditions: The ALECS cross-sectional study. Int. J. Behav. Nutr. Phys. Act..

[34] Hill T.D., Uchino B.N., Eckhardt J.L., Angel J.L. (2016). Perceived social support trajectories and the all-cause mortality risk of older Mexican American women and men. Res. Aging.

[35] Warren-Findlow J., Laditka J.N., Thompson M.E., Laditka S.B. (2013). Effects of social ties on self-rated physical health among African American adults. J. Natl. Med. Assoc..

[36] Tomini F., Tomini S.M., Groot W. (2016). Understanding the value of social networks in life satisfaction of elderly people: A comparative study of 16 European countries using SHARE data. BMC Geriatr..

[37] Garay S., Montes de Oca V., Guillén J. (2014). Social support and social networks among the elderly in Mexico. J. Popul. Ageing.

